# Minimalist optical system for achromatic imaging within extended field of view based on monolithic integrated meta-axicon cluster

**DOI:** 10.1038/s41377-026-02272-y

**Published:** 2026-04-16

**Authors:** Jianli Wang, Chengmiao Wang, Bin Wang, Yongting Deng, Yu Lin, Yeming Han, Lu Yao, Long Zhang, Dayu Li, Dejia Meng, Xiufeng Liu, Xiyu Li, Jan G. Korvink, Yongbo Deng

**Affiliations:** 1 Changchun Institute of Optics, Fine Mechanics and Physics, Chinese Academy of Sciences, Changchun, 130033 China; 2 Beijing Institute of Tracking and Telecommunications Technology, Beijing, 100094 China; 3 National Key Laboratory of Space Integrated Information System, Beijing, 100094 China; 4 Institute of Microstructure Technology (IMT), Karlsruhe Institute of Technology(KIT), Karlsruhe, 76344 Germany

**Keywords:** Optoelectronic devices and components, Metamaterials, Sub-wavelength optics, Imaging and sensing

## Abstract

Achromatic metalenses face stringent aperture and numerical aperture (NA) constraints, which have become a key bottleneck in metasurface imaging. To this end, a novel achromatic imaging method was first proposed, utilizing the unique wideband consistency of diffracted Bessel spots combined with non-blind image restoration techniques. To address the off-axis aberration issue, off-axis achromatic meta-axicons with eccentric conical phases were further designed to convert oblique plane waves into wideband uniform off-axis Bessel beams. Ultimately, a single metasurface integrating 9 meta-axicons with different design field angles was developed, and a meta-camera was constructed accordingly. After image restoration, the meta-camera achieves achromatic imaging within a 10° stitched field of view (FOV), and an angular resolution close to that of near-diffraction-limit lens with the same aperture throughout the entire FOV. The core idea of achieving achromatic imaging based on natural dispersion laws in this study enables the wideband minimalist optical system based on metasurface to completely circumvent the aperture limitation, providing a highly valuable solution for large-aperture meta-camera design that can simultaneously accommodate wideband and off-axis FOV.

## Abstract (web summary)

Minimalist optical system for achromatic imaging within extended field of view based on monolithic integrated meta-axicon cluster

## Graphical abstract

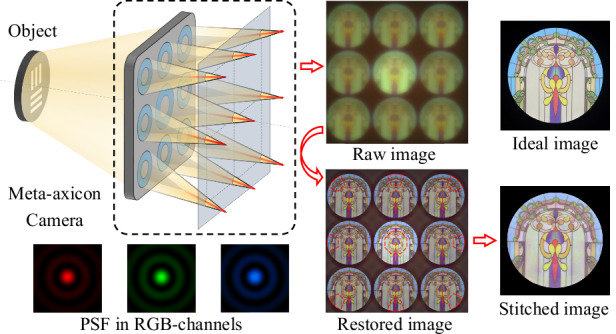

## Introduction

Metalenses, which manipulate light fields via subwavelength micro/nanostructures, have garnered significant research interest owing to their unique advantages including lightweight, easy integration and multi-dimensional wavefront manipulation capabilities^1–4^. They have enabled technological innovations in various fields such as LiDAR^5,6^, polarization imaging^7–9^, computational-generated holography^10–12^, near-eye displays^13,14^, biological microscopy^15–18^, and astronomical observations^19–21^. However, chromatic aberration remains a major constraint limiting their wideband applications. The most prevalent method for achieving achromatic functionality in metalenses is phase dispersion engineering, which involves the meticulous design of nanostructure cross-sectional morphologies within meta-atoms to realize both phase matching and phase dispersion matching^22–25^. The emergence of this method theoretically endows metalenses with achromatic capabilities. However, the phase dispersion generated by meta-atoms cannot achieve significant breakthroughs in magnitude within the current micro-nano fabrication technology framework, resulting in stringent mutual constraints among the aperture, NA, and bandwidth of achromatic metalenses^26^. Therefore, the apertures of most existing achromatic metalenses are on the order of hundreds of wavelengths, which fails to meet the requirements for most practical applications.

Currently, it has become a consensus that strict achromatism via phase matching within continuous spectra is nearly unattainable for metalenses with aperture sizes exceeding ten-thousand wavelengths. In this context, various innovative structural design methods have been proposed to achieve non-strictly achromatic performance, such as frequency-domain coherence optimization^27^, asymptotic phase compensation^28^, dispersion matching of meta-atom and material layer^29^, and quasi-continuous spectrum achromatic methods^30^. These approaches demonstrate remarkable success in relaxing the fundamental trade-offs between aperture size, NA, and operational bandwidth, while delivering satisfactory wideband imaging performance. Nevertheless, some concomitant defects are inevitably introduced, such as compromised focusing efficiency and residual chromatic aberration. In terms of the selection of underlying structures, multi-level concentric-ring structures have been adopted to expand the achromatic performance boundaries of meta-atom array^27,31–33^. The non-binary structural height is highly beneficial for chromatic aberration suppression while maintaining focusing efficiency, but it poses higher requirements on micro-nano processing capabilities and the corresponding specific optimization algorithms.

To address the achromatic challenge in large-aperture metalenses, this work proposes an alternative imaging paradigm that prioritizes the characteristics of wideband consistency of the point spread function (PSF) over conventional high-efficiency point focusing, which provide great convenience for terminal image restoration. Based on this idea, meta-axicons are selected as the core element for achromatic imaging. Their unique property is that the relative intensity distribution of the generated Bessel beam is independent of wavelength under the constraints of the grating equation, forming an achromatic effect that relies on natural dispersion. Scholars have compared metasurfaces with different phase forms, such as axicon phase^34,35^, cubic phase^36–38^, log-asphere phase^34,39^, and SQubic phase^40^ and summarized the performance differences in achieving wideband computational imaging through focal depth expansion using different phase distributions^41^. However, it is still in its infancy to conduct targeted research on meta-axicon-based achromatic computational imaging from the perspective of wideband-consistent PSF^42^. Moreover, the quality of Bessel spots generated by meta-axicons exhibits strong angular dependence, which constrains their effective FOV. To mitigate this limitation, off-axis meta-axicon configurations have been developed to enhance FOV coverage. By incorporating lateral chromatic aberration correction, these off-axis meta-axicons can effectively transform obliquely incident visible light into off-axis quasi-Bessel beam within a defined field angle range.

The final design integrates a monolithic metasurface comprising a 4 mm-diameter main meta-axicon surrounded by eight 3 mm-diameter off-axis meta-axicons. Each meta-axicon produces achromatic images for distinct FOV regions, and non-blind deconvolution image restoration based on the Total Variation (TV) regularization method is performed. The resulting multiple local fields are stitched into a 10° full FOV. This study breaks away from the traditional constraint of the achromatic design through phase dispersion control, enabling a significant increase in aperture size. Benefiting from the wideband consistency shared by both on-axis and off-axis Bessel spots, the restored images achieve a limiting angular resolution no less than 80% of that provided by conventional diffraction-limited lenses with equivalent apertures across the entire FOV. In terms of executability, this method also boasts advantages such as easy processing of micro-nano structures and rapid image restoration, providing a practical and feasible technical solution for the development of minimalistic metasurface optical systems capable of large-aperture achromatic imaging. For a performance comparison with other typical works on achromatic metasurface, please refer to Supplementary Information S1.

## Results

### Basic theory and method

**Fig. 1:**
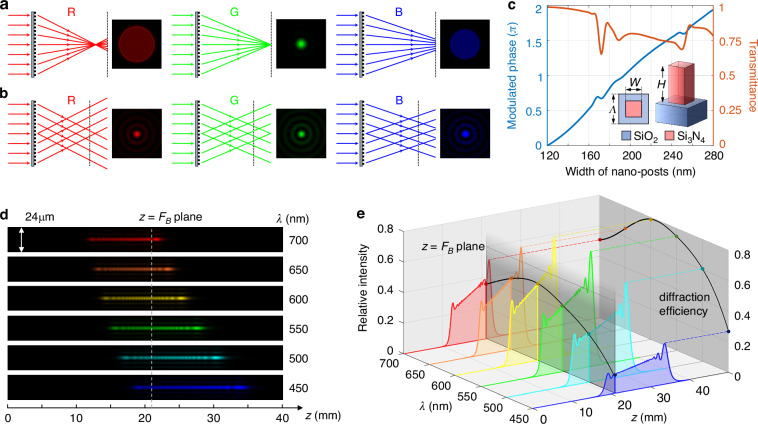
Schematic principle of meta-axicons for wideband-consistent PSF generation. **a** Ray tracing and PSF illustration of monochromatic metalenses; **b** Ray tracing and PSF illustration of meta-axicons; **c** Modulation phase and transmittance curves of meta-atoms, with embedded diagrams showing top and 3D views of the meta-atoms, having the central wavelength *λ*_0_ = 532 nm, the lattice period *Λ*=400 nm and the height *H* = 800 nm; **d** Simulation illustration of the cross-sectional intensity of the Bessel beam generated by the meta-axicon; **e** Axial relative intensity distribution, diffraction efficiency, and central intensity within the working plane of the wideband Bessel beam

The target phase of the axial near-diffraction-limit focusing metalens is in the form of hyperboloid, as shown in Eq. (1). Here, *r*, *λ*, *F* represent radial coordinate, wavelength and focal length, respectively, and *C*(*λ*) is a phase constant independent of spatial coordinate. Current metalens design methods based on phase dispersion engineering struggle to achieve wideband phase matching of Eq. (1), and metalenses designed only for the central wavelength *λ*_0_ exhibit severe chromatic aberration, with the focal length offset proportional to −Δ*λ*/(*λ*_0_ + Δ*λ*) ⋅ *F* under small NA, as illustrated in Fig. 1a. This chromatic aberration, mixed with off-axis aberrations carried by the hyperbolic phase, poses significant challenges for high-quality image restoration.

1 $$\varPhi \left(r,\lambda \right)=-\frac{2\pi }{\lambda }\left(\sqrt{{r}^{2}+{F}^{2}}-F\right)+C(\lambda )$$

In contrast, the target phase of meta-axicon used to generate a 0th-order Bessel beam is shown in Eq. (2), and the complex amplitude at the distance *z* is given by Eq. (3). Here, *α*_0_ is the aperture angle of the meta-axicon at the central wavelength *λ*_0_, *A* is a scaling factor, *k*_*z*_ = 2π/*λ*⋅cos*α* and *k*_*r*_ = 2π/*λ*⋅sin*α* are the axial and radial wave vectors, respectively, and *J*_0_ is the 0th-order Bessel function.

2 $$\varPhi \left(r\right)=-\frac{2\pi }{{\lambda }_{0}}r\cdot sin{\alpha }_{0}$$

3 $$E(r,\,z,\,\lambda )=A\cdot \exp (i{k}_{z}\cdot z)\cdot {J}_{0}({k}_{r}\cdot r)$$

For a meta-axicon with a radial structural period *P*, the aperture angle of the transmitted light is determined by the 1st-order diffraction grating equation *P*⋅sinα = *λ*. Substituting this into Eq. (3) yields Point Spread Function (PSF) of the wideband Bessel beam as *A*^2^·*J*_*0*_^2^(2π/*P*·*r*), which does not contain the wavelength variable except for the overall scaling factor *A*, indicating that the relative intensity of the Bessel spot generated by the meta-axicon exhibits excellent wideband consistency, as shown in Fig. 1b.

After clarifying the achromatic advantage of Bessel spots, the nano structure design of the meta-axicon was conducted. Figure 1c shows the modulation phase and transmittance curves of the meta-atoms, with the lattice period of *Λ* = 400 nm, the height of nano-pillars of *H* = 800 nm, and the cross-sectional dimensions ranging from 120 nm to 280 nm, ensuring 2π phase modulation at the central wavelength of *λ*_0_ = 532 nm. Subsequently, a meta-axicon with a diameter of *D*_1_ = 4 mm and a focal length of *F*_0_ = 30 mm was arranged based on the constructed building block library. Accordingly, the aperture angle is *α*_0_ = 3.81°, represents a trade-off between the full width at half maximum (FWHM) and the field angle sensitivity of the PSF. Considering that the region with *r* < *D*_1_/4 in the meta-axicon does not contribute to the generation of Bessel spots, this region is designed as a light-blocking structure to reduce the interference of 0th-order diffraction on the final imaging. Within the working bandwidth of 450 nm to 700 nm, the simulated intensity of the generated Bessel beam is shown in Fig. 1d, and the diffraction efficiency and the central intensity at the back focal plane are shown in Fig. 1e. Thus, the effective interval of Bessel beam with a wavelength *λ* is *Z*_*λ*_ = [*λ*_0_*F*_0_/2*λ*, *λ*_0_*F*_0_/*λ*], and the axial range of Bessel beam encompassing the entire design bandwidth is [*λ*_0_*F*_0_/2*λ*_min_, *λ*_0_*F*_0_/*λ*_max_]. In this work, the back working distance of *F*_*B*_ = 21 mm is selected to ensure the reception of the full-spectrum Bessel spot and achieve the largest possible focal depth. The central intensity of the Bessel spot in *z* = *F*_*B*_ plane will be used to guide the manual adjustment of white balance in subsequent imaging, determined by both the diffraction efficiency spectrum (the peak envelope in Fig. 1e) and the relative position of the back working distance *F*_*B*_ within the effective interval *Z*_*λ*_. For specific details on the simulation of Bessel beam intensity, please refer to Supplementary Information S2.

### Design of off-axis meta-axicons for FOV expansion

**Fig. 2:**
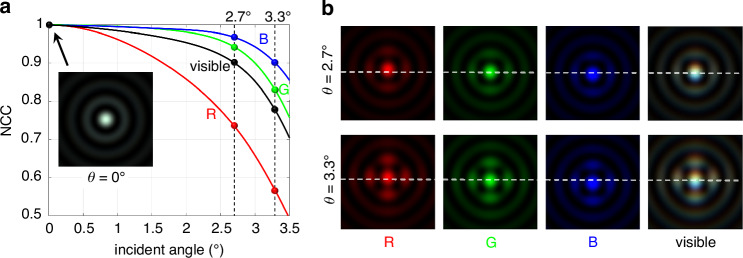
Schematic diagram of off-axis PSF degradation of the conventional meta-axicon. **a** Attenuation curve between NCC and incidence angle in different spectral bands, the embedded image is the on-axis PSF, with corresponding NCC = 1; **b** PSF illustrations at the maximum field angle (2.7°) and an unacceptable field angle 3.3°, with a simulation range of 24 μm × 24 μm for both

The above design results fully verify the wideband consistency of Bessel spots, but this conclusion theoretically holds strictly only under normal incidence conditions. Non-zero incidence angles will disrupt the circular symmetry of the output wavefront and introduce monochromatic off-axis aberrations and lateral chromatic aberrations. Regarding the changes in PSF, they are specifically manifested as the secondary diffraction rings enhancement along the meridional and sagittal directions, and the drift of the centroid position across the entire working bandwidth. These imperfections will seriously affect the large-field non-blind image restoration using the ideal Bessel spot as the convolution kernel. The Normalized Cross-Correlation (NCC) of the full-field PSF relative to the on-axis PSF was defined according to Eq. (4) to evaluate its field consistency, where *psf* and *psf*_0_ represent the intensity distributions of the off-axis and on-axis spots, respectively. The attenuation curve of NCC with field angle *θ* is shown in Fig. 2a, and the wideband PSFs under specific field angles are shown in Fig. 2b. It is assumed that NCC = 0.9 is the acceptable lower limit for spot degradation across the entire visible spectrum, and the corresponding incidence angle of 2.7° is the maximum field angle for image restoration. At this field position, the non-uniformity in sidelobe intensity becomes significant and the spot displacement caused by lateral chromatic aberration reaches the level of the FWHM of the main lobe.

4 $${\mathrm{NCC}}(\theta )=\frac{{\sum }_{x,y}({psf}-\bar{{psf}})\cdot ({{psf}}_{0}-\bar{{{psf}}_{0}})}{\sqrt{{\sum }_{x,y}{({psf}-\bar{{psf}})}^{2}\cdot {\sum }_{x,y}{({{psf}}_{0}-\bar{{{psf}}_{0}})}^{2}}}$$

**Fig. 3:**
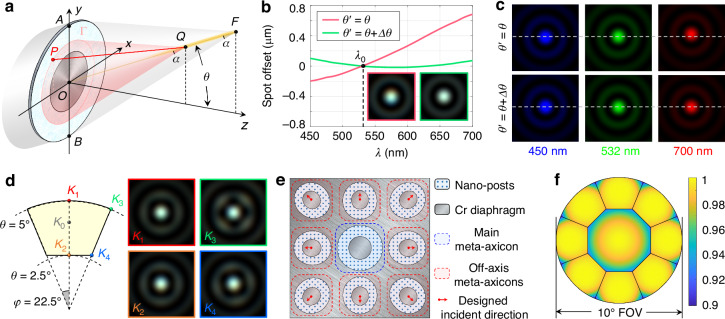
Schematic diagram of the design principle and arrangement method of the off-axis meta-axicons. **a** Phase solution schematic of the off-axis meta-axicon; **b** Curves of focal spot offset before and after wideband chromatic aberration correction; **c** PSFs at wavelengths *λ*_min_, *λ*_0_, and *λ*_max_ before and after correction; **d** Effective FOV of the off-axis meta-axicon and PSFs at the main boundary positions; **e** Schematic of a monolithic metasurface integrated with 9 meta-axicons, where the contour eccentricity of the off-axis meta-axicons is slightly exaggerated; **f** Effective FOV stitching schematic of the metasurface in (**e**) and its internal NCC value distribution

It can be seen that the wideband consistency of Bessel spots generated by conventional meta-axicons rapidly decreases with increasing incident angle, severely limiting the FOV that allows for high-quality image restoration. To overcome this shortcoming, we further meticulously designed off-axis meta-axicons for field expansion, tailored to meet the high-resolution image restoration requirements of off-axis points, as shown in Fig. 3a. Under the ray tracing approximation model, the rays incident obliquely to point P should propagate along the generatrix of the conical surface with the propagation direction of the Bessel beam as its axis and a constant angle *α* as its ‌semi-vertex angle after being deflected by the meta-axicon. Here, the propagation direction of the Bessel beam, namely the inclination angle of the cone axis, is initially assumed to be equal to the incidence angle *θ*. According to the beam deflection requirements and the 2D generalized Snell’s law, the phase gradient field within the effective aperture of the off-axis meta-axicon can be calculated. However, this gradient field determined by point-to-point ray tracing does not satisfy the irrotationality property and cannot be used to strictly recover the phase distribution. Simultaneously, the intensity of the Huygens wavelet radiated from each point on the meta-axicon no longer possesses circular symmetry for off-axis points. Therefore, theoretically, a single metasurface cannot strictly convert oblique incident light into a co-propagating Bessel beam, which is fundamentally different from the design of metalens for near-diffraction-limited focusing at off-axis points.

To this end, the strictness of the off-axis Bessel beam field is appropriately relaxed, and the principle for solving the target modulation phase is shifted from global phase gradient integration to local equal optical path constraint. In Fig. 3a, the vertex Q of the pink cone is any point on the off-axis Bessel beam, and the ‌semi-vertex angle *α*_0_ is the aperture angle of the off-axis Bessel beam at the central wavelength *λ*_0_. The cone intersects the *xoy*-plane where the meta-axicon is located, forming an ellipse Г. The Huygens wavelets emitted from all points on the ellipse Г should achieve coherent superposition at point Q. Therefore, the phase *Φ* at point P(x,y) can be solved using the equal optical path relationship shown in Eq. (5), where *F*_0_ is the distance from the off-axis focus F to the *xoy*-plane; K denotes the linear scaling factor of the two cones with vertices Q and F, respectively, that is, the length ratio of line segments OQ and OF.

5 $$\frac{2\pi }{{\lambda }_{0}}{ysin}\theta +\varPhi +\frac{2\pi }{{\lambda }_{0}}\sqrt{{x}^{2}+{(K{F}_{0}\tan \theta -y)}^{2}+{\left(K{F}_{0}\right)}^{2}} =\frac{2\pi }{{\lambda }_{0}}\frac{K{F}_{0}}{\cos \theta }\cos {\alpha }_{0}$$

The three terms on the left side of Eq. (5) are the tilt phase of the incident light, the modulated phase of the metasurface and the phase delay introduced by line segment PQ, respectively, while the right side is the product of the wave vector and the position vector at point Q, that is, the phase possessed by the off-axis Bessel beam as it propagates to point Q. Similar to the on-axis meta-axicon, the region with a scaling factor K less than 0.5 on the off-axis meta-axicon is replaced with a light stop to avoid undesired interference caused by 0th-order light leakage.

Based on the above phase solution method, the structural design of the off-axis meta-axicon for incident angle of *θ* = 4° was conducted. To ensure its applicability within a certain range of FOV, the aperture angle α_0_ at the central wavelength was reduced to 2.86°, corresponding to the aperture angle of an axicon with a diameter of *D*_2_ = 3 mm and a focal length of *F* = 30 mm. Simulation results show that the PSF of the non-strict Bessel beam remains highly consistent with the standard Bessel beam but exhibits a small amount of lateral chromatic aberration. To address this issue, a wideband constraint was further introduced based on the aforementioned design method, aiming to balance the lateral chromatic aberration that was not considered during the process of monochromatic structure design. Specifically, a small amount of tilt along the meridian direction was superimposed on the original phase distribution, making the propagation angle of Bessel beam *θ‘* at the central wavelength different from the incident angle *θ*. The optimal *θ‘* value should ensure that its mean square error *δ* is minimized within the entire spectrum. In Fig. 3a, *θ*_*A*_(*λ*) and *θ*_*B*_(*λ*) are defined as the propagation angles of the outgoing rays at points on line segments OA and OB in *yoz*-plane, respectively, and the propagation angle of the Bessel beam is [*θ*_*A*_(*λ*) + *θ*_*B*_(*λ*)]/2. Thus, the optimization problem regarding *θ‘* can be expressed by Eq. (6).

6 $$\mathrm{Min}\,\delta =\frac{1}{{\lambda }_{\max }-{\lambda }_{\min }}{\int }_{{\lambda }_{\min }}^{{\lambda }_{\max }}{\left[\frac{1}{2}\left({\theta }_{A}\left(\lambda \right)+{\theta }_{B}\left(\lambda \right)\right)-{\theta }^{\mathrm{'}}\right]}^{2}{\rm{d}}\lambda$$

where *θ*_*A*_(*λ*) and *θ*_*B*_(*λ*) satisfy the proportional relationship determined by the grating equation, as shown in Eq. (7):

7 $$\frac{\sin {\theta }_{A}\left(\lambda \right)-\sin \theta }{\sin \left({\theta }^{\mathrm{'}}-{\alpha }_{0}\right)-\sin \theta }=\frac{\lambda }{{\lambda }_{0}},\frac{\sin {\theta }_{B}\left(\lambda \right)-\sin \theta }{\sin \left({\theta }^{\mathrm{'}}+{\alpha }_{0}\right)-\sin \theta }=\frac{\lambda }{{\lambda }_{0}}$$

The solution to Eq. (6) shows that the optimal Δ*θ*, the difference between *θ‘* and *θ*, is -0.105mrad. Since *θ‘* is known, replace *θ* in all positions except the first term representing incident tilt in Eq. (5) with *θ‘*, and the phase distribution *Φ* under the constraint of lateral chromatic aberration can be solved. By comparing the quality of the wideband Bessel beam before and after correcting the small angle, we obtained the lateral chromatic aberration represented by the curve of focal spot offset, as shown in Fig. 3b, with the inset showing the corresponding full-color PSF. Figure 3c shows the PSFs at wavelengths *λ*_min_, *λ*_0_, and *λ*_max_ before and after correction. It is evident that the focal spot shift, which measured nearly 1μm prior to correction, is reduced to under 100 nm after correction, demonstrating rigorous control over lateral chromatic aberration. For specific details on the phase solution and lateral chromatic aberration correction of the off-axis meta-axicon, please refer to Supplementary Information S3.

The effective FOV of the off-axis meta-axicon can be estimated, given that its wideband performance has been verified at the designed incident angle. Due to the absence of circular symmetry, the acceptable range of the field angle of the off-axis meta-axicon will be smaller than that of the main meta-axicon. Here, NCC ≥ 0.9 was still adopted as the criterion for the effective FOV. Consequently, the effective FOV is delineated as the region where the inclination angle falls between 2.5° and 5°, and the azimuth angle is less than 22.5°. The PSFs at the edge positions are shown in Fig. 3d. Ultimately, 8 off-axis meta-axicons rotated by different azimuth angles and the main meta-axicon are integrated into a monolithic metasurface, enabling achromatic imaging with a full effective FOV expanded to 10°, as illustrated in Fig. 3e, with the corresponding NCC pseudocolor map of the FOV space shown in Fig. 3f.

### Image Processing for Meta-Axicon Imaging

The non-blind deconvolution image restoration module in this paper is constructed based on the multi-order diffraction of the meta-axicon. The wideband mean values of the diffraction efficiency for each diffraction order exhibit a rough magnitude relationship: ${\bar{\eta }}_{1}\gg {\bar{\eta }}_{0}\gg {\bar{\eta }}_{{else}}$. Therefore, only the 0th and 1st diffraction orders of the metasurface are considered during the development of the image restoration module. Thanks to the annular diaphragm structure design, the intensity distributions of the 0th and 1st diffraction orders on the imaging plane can be completely separated in space, and the overall PSF can be written as the sum of their intensities even if they are not incoherent light. For detailed simulation analyses of multi-order diffraction efficiencies and global PSFs, please refer to Supplementary Information S4. The convolution model for imaging within a fixed imaging band [*λ*_min_, *λ*_max_] by the metasurface axicon can be written as Eq. (8):

8 $$Y={\int }_{{\lambda }_{\min }}^{{\lambda }_{\max }}{\eta }_{1}\left(\lambda \right)\cdot \,{{psf}}_{\left(1\right)}\left(\lambda \right)* I\left(\lambda \right)d\lambda+{\int }_{{\lambda }_{\min }}^{{\lambda }_{\max }}{\eta }_{0}\left(\lambda \right)\cdot \,{{psf}}_{\left(0\right)}\left(\lambda \right)* I\left(\lambda \right)d\lambda +{\rm{\varepsilon }}$$

where *Y* represents the intensity value of any RGB channel in the directly imaged results, and the two terms on the right side are the intensity contributions of the 1st and 0th diffraction orders, respectively. *I*(*λ*) is the intensity spectrum of the ideal image, ‘*‘ is the two-dimensional convolution operator defined on the imaging plane, and *ε* is noise. *η*_*m*_(*λ*) and *psf*_(*m*)_(*λ*) are the diffraction efficiency and point spread function of the *m*th diffraction order, respectively, where *m* = 1 or 0. The two-dimensional integrals of *psf*_(*m*)_(*λ*) on the imaging plane are normalized to 1. This image restoration problem can be stated as solving *I*(*λ*) given *Y*, *η*_*m*_(*λ*), and *psf*_(*m*)_(*λ*). If the spatial distribution of *psf*_(*m*)_(*λ*) strongly depends on *λ* (e.g., for monochromatically designed metalenses), then the known quantities in this problem will be far fewer than the unknown quantities, making it difficult to solve the image quality degradation caused by significant chromatic aberration using image processing techniques.

The key to solving this problem lies in making the spatial distribution of the PSF as consistent as possible, even if its far-exceeding energy divergence of the Airy disk affects the direct imaging quality. This core requirement for the PSFs leads us to choose Bessel beam for imaging. Although from the perspective of large-scale energy divergence, the effective regions of Bessel light at different wavelengths are not the same, the energy distribution across the RGB tri-channels does not exhibit significant trend changes within the full visible spectrum, as indicated by the intensity distributions in Fig. S3. Therefore, *psf*_(1)_(*λ*) can approximately satisfy the design requirement of wideband consistency within each channel. For the directly transmitted 0th diffraction order, its light intensity distribution can be approximated as the pupil function, which obviously satisfies the requirement of wideband consistency. As the only two diffraction orders of the meta-axicon that satisfy wideband consistency, the actual image produced by the convolution of the two with *I*(*λ*) can be rewritten as Eq. (9).

9 $${l}Y={{psf}}_{\left(1\right)}* {\int }_{{\lambda }_{\min }}^{{\lambda }_{\max }}{\eta }_{1}\left(\lambda \right)\cdot \,I\left(\lambda \right)d\lambda+{{psf}}_{\left(0\right)}* {\int }_{{\lambda }_{\min }}^{{\lambda }_{\max }}{\eta }_{0}\left(\lambda \right)\cdot \,I(\lambda )d\lambda +{\rm{\varepsilon }}$$

Based on this, the average diffraction efficiencies within the band [*λ*_min_, *λ*_max_] are extracted to further simplify Eq. (9), resulting in Eq. (10):

10 $$Y={\bar{\eta }}_{1}{{psf}}_{\left(1\right)}* {\int }_{{\lambda }_{\min }}^{{\lambda }_{\max }}I\left(\lambda \right)d\lambda +{\bar{\eta }}_{0}{{psf}}_{\left(0\right)}* {\int }_{{\lambda }_{\min }}^{{\lambda }_{\max }}I(\lambda )d\lambda +{\rm{\varepsilon }}$$

The further approximation in Eq. (10) is reasonable because the quantum efficiency of actual detectors also varies at different wavelengths within a wide band. Their detection results essentially represent the intensity spectrum integral weighted by the quantum efficiency spectrum, which is used to approximately replace the unweighted intensity spectrum integral. The actual imaging model in this work is equivalent to applying an additional correction to the quantum efficiency spectrum using the diffraction efficiency spectrum, still following the basic principle of energy detection.

From this point on, the PSF of this imaging system can be regarded as ${\bar{\eta }}_{1}{{psf}}_{(1)}+{\bar{\eta }}_{0}{{psf}}_{\left(0\right)}$, which also possesses the wideband consistency of relative intensity distribution. ${\int }_{{\lambda }_{\min }}^{{\lambda }_{\max }}I(\lambda )d\lambda$ can be obtained using non-blind image restoration methods. The TV regularization method^43,44^ is employed to optimize the quality of the reconstructed images for each RGB channel individually. The objective function is defined as Eq. (11):

11 $$\hat{\beta }=\mathop{{\rm{argmin}}}\limits_{\beta }{\rm{||}}{\left({\,\bar{\eta }}_{1}{{psf}}_{\left(1\right)}+{\bar{\eta }}_{0}{{psf}}_{\left(0\right)}\right)* \beta -Y}{\rm{||}^{2}}+\mu \cdot {TV}(\beta )$$

where $\beta ={\int }_{{\lambda }_{\min }}^{{\lambda }_{\max }}I(\lambda )d\lambda ,TV(\beta )={\sum }_{i}{\Vert {D}_{i}\beta \Vert }_{2}$. *D*_*i*_ denotes the discrete gradient operator at pixel *i*, which extracts the local gradient vector (typically the forward or central finite differences in horizontal and vertical directions), and *TV*(*β*) represents the sum over all pixels of the norm of the discrete gradient vector, referred to as the TV regularization term, and *μ* denotes the regularization coefficient. The introduction of the total variation regularization term promotes smoothness in the image and aids in preserving edge details.

### Fabrication and Performance Testing of Meta-Axicon Cluster

**Fig. 4:**
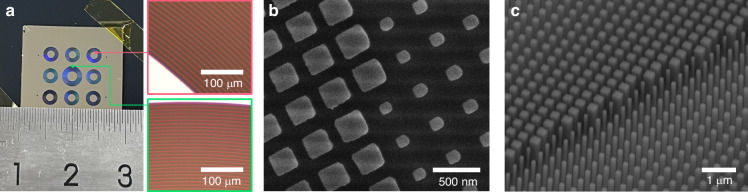
The processing result of the monolithic integrated meta-axicon cluster. **a** Physical image of the metasurface sample integrated with 9 meta-axicons and optical microscopic images of local positions; **b** Local top-view electron microscopic image; **c** Local oblique-view electron microscopic image

Based on the aforementioned design, Electron Beam Lithography (EBL) technology was utilized for the fabrication of the metasurface sample, and the morphological characterization results of the nanostructures are presented in Fig. 4. The detailed process flow is provided in Supplementary Information S5.

**Fig. 5:**
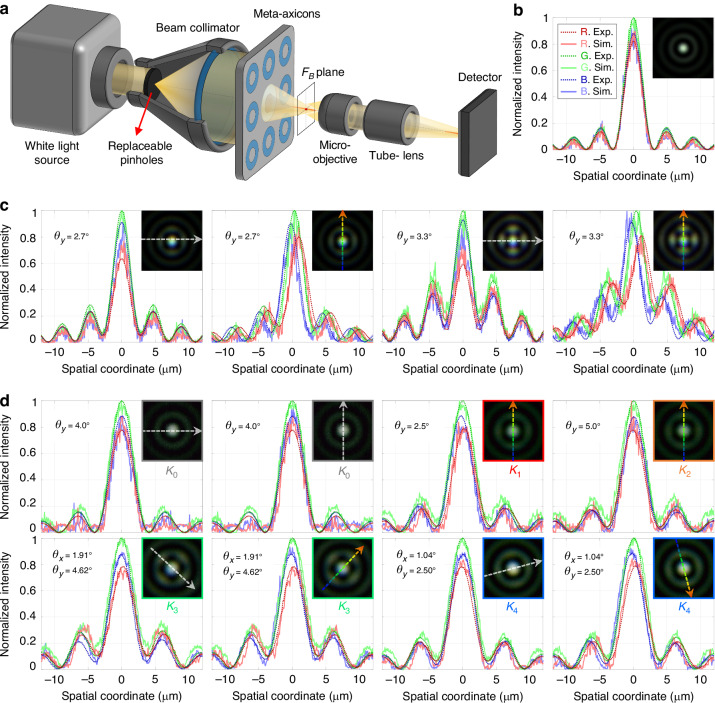
The PSF test results of the meta-axicons. **a** Schematic diagram of the optical system for PSF detection of meta-axicons; **b**–**d** Intensity profile and overall image of the PSF of **b** main meta-axicon under normal incidence; **c** main meta-axicon under oblique incidence; **d** off-axis meta-axicon under different FOV positions. The omitted legends of (**c**) and (**d**) are the same as that of (**b**). The intensity profiles in (**c**) and (**d**) are recorded along the directions of the arrows in the insets, with the color arrows and the gray arrows representing the dispersion directions and their perpendicular directions, respectively

After fully verifying that the structural morphology of the fabricated metasurface sample matches the design results, an optical system for PSF testing was first set up, as shown in Fig. 5a. A white light source is filtered, expanded, and collimated to emit a large-aperture plane wave, which generates a Bessel beam after passing through the meta-axicon. A 40× magnified microscopic system composed of a microscope objective, a tube lens, and a scientific camera (TUCSEN Co., LTD, Dhyana 401D, pixel size 6.5μm, pixel scale 2048×2048) is used for RGB tri-channel intensity acquisition and white balance calibration. Figure 5b shows the radial intensity profile of the on-axis Bessel spot, with the inset showing the corresponding full-color imaging result. The measured FWHM in RGB channels are 2.89 μm, 2.87 μm, and 2.85 μm, respectively, which are highly close to the constant FWHM of the standard Bessel spot, 0.36*λ*_0_/sin*α*_0_, demonstrating high consistency of the wideband PSF in terms of relative intensity distribution. As the collimated light is obliquely incident on the main meta-axicon at angles of *θ*_*y*_ = 2.7° and *θ*_*y*_ = 3.3°, the secondary diffraction rings of the Bessel spot exhibit undesired intensity enhancements in both meridional and sagittal directions due to the presence of monochromatic off-axis aberrations, while lateral chromatic aberrations further introduce an offset along *y*-direction for the spots within the RGB tri-channels, as shown in Fig. 5c. In particular, the PSF at *θ*_*y*_ = 3.3° has severely deviated from the assumption of globally consistent PSF, demonstrating the necessity of utilizing the off-axis meta-axicon instead of main meta-axicon to perform imaging tasks with a large FOV.

The PSFs of the off-axis meta-axicons at several primary FOV positions are shown in Fig. 5d. At the design field angle *θ*_*y*_ = 4° (point *K*_0_ in Fig. 3d), the PSF of the off-axis meta-axicon is almost indistinguishable from the intensity distribution of the standard Bessel spot, demonstrating excellent achromatic properties. At the meridian FOV boundary (points *K*_1_ and *K*_2_ in Fig. 3d), the slight chromatic aberration along y-direction and the intensity fluctuations of secondary diffraction rings can be ignored. Their intensity profiles along x-direction are omitted from Fig. 5d due to essential agreement with that at point *K*_0_. The PSF deterioration at point *K*_3_ is similar to that at the maximum field angle *θ*_*y*_ = 2.7° of the main meta-axicon, reaching the tolerance limit for global PSF deterioration, which is consistent with the spot quality evaluation results based on NCC in Fig. 3f.

In addition, since the image restoration model in this paper is established based on the globally divergent PSF, the global intensity distribution of the diffraction field has also been carefully tested and verified. For specific details on global PSF testing, please refer to Supplementary Information S4.

**Fig. 6:**
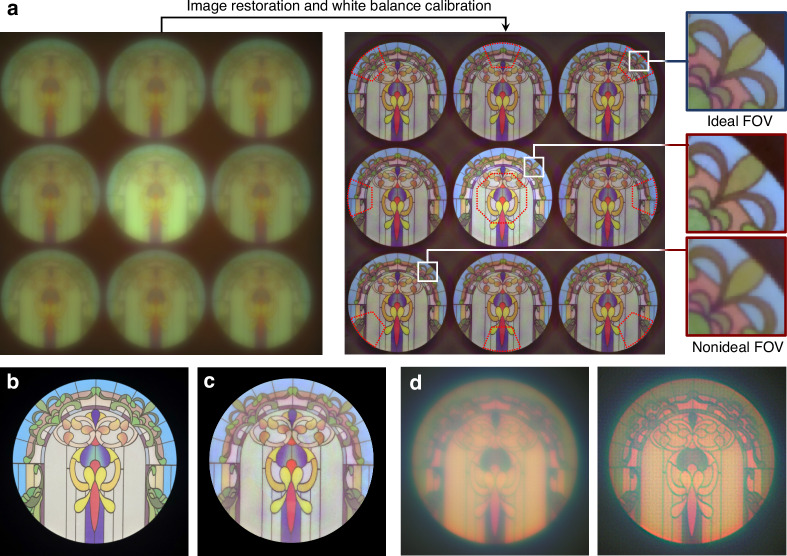
Imaging results of the meta-axicon cluster on a color target with a full field angle of 10°. **a** Direct imaging results, global image restoration results, and local zoomed-in figure; **b** Reference object; **c** Extraction and stitching results of high-quality restored regions; **d** Direct imaging and restoration results of a monochromatic metalens with the same aperture and focal length

After verifying that the point focusing ability of the meta-axicon cluster meets expectations within the entire design FOV, its joint imaging ability was officially tested. The monolithic metasurface and detector were packaged into a minimalistic optical system, which was then used to image a 35-cm-diameter circular pattern positioned ~2 m from the camera with a full FOV of 10°. The direct imaging results and global image restoration results are shown in Fig. 6a. The PSFs of the main meta-axicon and off-axis meta-axicons differ in absolute intensity and FWHM due to the difference in their apertures (4 mm for main meta-axicon and 3 mm for off-axis meta-axicons), while their diffraction efficiencies and relative spectral intensity distributions are essentially the same. Therefore, image restoration based on non-blind deconvolution and brightness correction are carried out separately for on-axis and off-axis images, while white balance calibration is performed uniformly for the overall image. There are non-negligible differences in image restoration details as evidenced by the zoomed-in figure of the same position in 9 images, confirming that the image restoration based on the assumption of full-field consistency of the PSF can only output high-quality image within the neighborhood of the designed field angle. 9 high-quality restored regions (outlined with red dotted lines in the image restoration results in Fig. 6a) were extracted and stitched into a whole, as shown in Fig. 6c. In comparison to the reference object depicted in Fig. 6b, the restoration results exhibit excellent resolution and contrast. Moreover, to compare the differences in wideband imaging effects, this work also constructed a monochromatic metalens with the same aperture (4 mm) and focal length (21 mm) based on the aforementioned building block library. The imaging results exhibit more pronounced chromatic aberration, and the strong spectral sensitivity of the PSF makes it difficult to be perfectly corrected by the backend computational optics module, as shown in Fig. 6d.

**Fig. 7:**
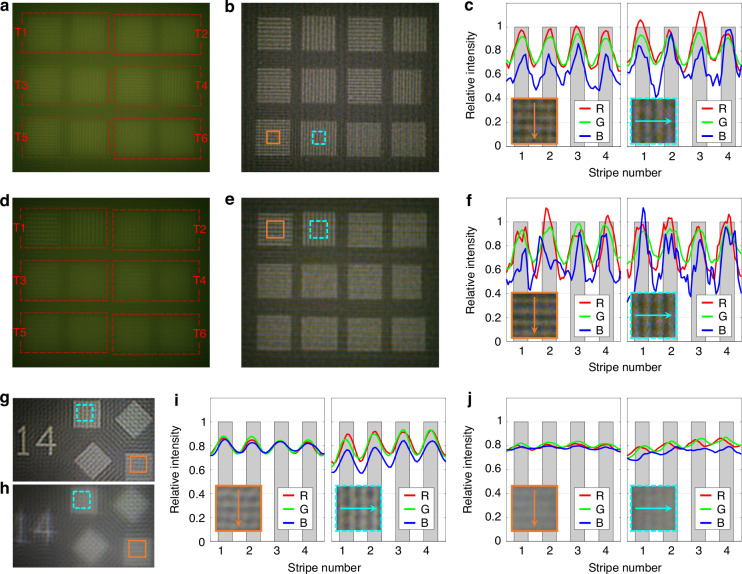
Imaging results of the meta-axicons on different resolution targets. **a**–**c** The direct imaging results, restoration results, and stripe relative intensity of the on-axis resolution target by the main meta-axicon, respectively; **d**–**f** The direct imaging results, restoration results, and stripe relative intensity of the resolution target positioned at the design incidence angle of 4° by the off-axis meta-axicon, respectively; **g**, **h** The ultimate resolution effect of the main meta-axicon on small FOV targets at incidence angles of 0° and 2.7°, respectively; **i**, **j** The stripe relative intensities in (**g**) and (**h**), respectively

While completing the overall imaging performance test, further measurements were conducted on the ultimate resolution capability of the meta-camera. The 40× microscopic system in Fig. 5a remained in use for conducting the resolution target imaging experiments to avoid the influence of the finite pixel size of the detector. Details of the tested resolution target are provided in Supplementary Information S6. The direct imaging results and restoration results of the on-axis resolution target through the main meta-axicon are shown in Fig. 7a, b, respectively. The resolvable limit in Fig. 7b is target T5, with an angular resolution of 5.13 lp/mrad, and its stripe relative intensity is shown in Fig. 7c. The direct imaging results and restoration results of the resolution target positioned at a design angle of 4° through the off-axis meta-axicon are shown in Fig. 7d, e, respectively. The resolvable limit in Fig. 7e is target T1, with an angular resolution of 3.76 lp/mrad, and its stripe relative intensity is shown in Fig. 7f. Correspondingly, the angular resolutions of near-diffraction-limited lenses with the same apertures as the two meta-axicons are *D*_1_ /1.22*λ*_0_ = 6.16 lp/mrad and *D*_2_ /1.22*λ*_0_ = 4.62 lp/mrad, respectively. The angular resolution limits of both types of meta-axicons in this study can achieve over 80% of that of a lens with the same aperture, with a resolution contrast ratio of no less than 0.2, thereby preliminarily validating the effectiveness and rationality of the achromatic imaging method proposed herein.

Our experimental investigation reveals that the limiting resolution of the meta-axicon imaging system exhibits weak dependence on FOV range and sparsity of the light intensity distribution of the observed target. Although the image restoration module can achieve nearly uniform limiting resolution for observed targets with different FOV ranges and light intensity distributions in an ideal noise-free model. In actual shooting processes, the influences of detector noise and edge field effects on the image restoration effect cannot be completely avoided, resulting in a negative correlation between the actual limiting angular resolution and the observed FOV range. Therefore, the limiting angular resolution test was repeated using the main meta-axicon for isolated targets with a FOV range of ~0.2°. The processed images at incidence angles of 0° and 2.7° are shown in Fig. 7g, h, respectively, and the corresponding stripe relative intensity distributions are shown in Fig. 7i, j, respectively. The angular resolution of the target in Fig. 7g is 6.61 lp/mrad, which is already close to the limiting angular resolution limited by the FWHM of the Bessel spot, *F*_*B*_/(0.36 *λ*_0_ /sin*α*_0_) = 7.29 lp/mrad, slightly exceeding the limiting angular resolution of a lens with the same aperture and focal length, 6.16 lp/mrad. This result indicates that the meta-axicon imaging system exhibits superior noise immunity when observing isolated targets within small FOVs due to the reduction of PSF sidelobe aliasing. Although there are still diffuse false bright stripes in the processed images, this does not prevent the overall system from possessing a resolution not inferior to that of traditional optical lens groups with the same aperture. Meanwhile, the decreased resolution of the main meta-axicon for isolated targets at the field angle of 2.7° in Fig. 7h, j once again illustrates the necessity of off-axis meta-axicons in expanding the range of high-resolution FOV.

Furthermore, the extended-depth-of-field characteristic, serving as a secondary advantage of using Bessel beams for achromatic imaging, has been validated in our experiments. Details are provided in Supplementary Information S7.

## Discussion

This work proposes a minimalistic optical system design method for achromatic imaging using a monolithic metasurface integrated with multiple meta-axicons. The method leverages the wideband consistency of the relative intensity distribution of zero-order Bessel spots under natural dispersion conditions to generate globally achromatic images. Meanwhile, a non-blind deconvolution computation module compatible with it has been developed to achieve high-definition image restoration. To further address the deficiencies of conventional meta-axicons, which are sensitive to off-axis aberrations and severely limited in FOV, off-axis meta-axicons were specifically designed to convert oblique incident light into Bessel beam without off-axis aberrations and lateral chromatic aberrations, aiming to expand the FOV of high-quality imaging. Ultimately, a monolithic metasurface composed of 1 main meta-axicon with a diameter of 4 mm and 8 off-axis meta-axicons with diameters of 3 mm was fabricated, achieving achromatic imaging within a 10° stitched FOV and reaching a limiting angular resolution of no less than 80% of that of a lens with the same aperture. The design of above meta-axicon cluster effectively overcomes the limitations of monolithic metasurface imaging in terms of aperture, FOV, and wideband resolution, significantly promoting the development of minimalist optical systems based on metasurfaces.

In subsequent research, wideband computational imaging with better noise resistance and color authenticity can be further achieved by integrating the presented meta-axicon cluster with sidelobe suppression techniques based on high-order vortex Bessel beams^42^ and improving the wideband chromatic uniformity via meta-atom optimization.

## Materials and methods

In this paper, a dielectric metasurface made of silicon nitride was fabricated on a quartz glass substrate, and a chromium film was integrated onto the metasurface to serve as the diaphragm of the meta-axicons. The micro-nano fabrication process utilizes electron beam lithography combined with etching to achieve the structuring of dielectric materials. For detailed information on the preparation of metasurfaces, please refer to Supplementary Information S5.

## Supplementary information

Supplementary information: Minimalist optical system for achromatic imaging within extended field of view based on monolithic integrated meta-axicon cluster

## Data Availability

Data underlying the results presented in this paper are not publicly available at this time but may be obtained from the authors upon reasonable request.
