# Supplementary material for: Minimalist optical system for achromatic imaging within extended field of view based on monolithic integrated meta-axicon cluster
Source: Light Sci Appl. 2026 Apr 16;15:202. doi: 10.1038/s41377-026-02272-y (PMC13087133; doi:10.1038/s41377-026-02272-y)
Supplement: Supplementary file 1 — Supplementary information: Minimalist optical system for achromatic imaging within extended field of view based on monolithic integrated meta-axicon cluster [file 41377_2026_2272_MOESM1_ESM.docx]

**Supplementary information：Minimalist optical system for achromatic imaging within extended field of view based on monolithic integrated meta-axicon cluster**

Jianli Wang^1^, Chengmiao Wang^1,*^, Bin Wang^1,*^, Yongting Deng^1,*^, Yu Lin^1^, Yeming Han^1^, Lu Yao^1^, Long Zhang^1^, Dayu Li^1^, Dejia Meng^1^, Xiufeng Liu^1^, Xiyu Li^2,3^, Jan G. Korvink^4,*^, Yongbo Deng^4,*^

*^1^Changchun Institute of Optics, Fine Mechanics and Physics, Chinese Academy of Sciences, Changchun, 130033, China*

*^2^Beijing Institute of Tracking and Telecommunications Technology, Beijing, 100094, China*

*^3^National Key Laboratory of Space Integrated Information System. Beijing, 100094, China*

*^4^Institute of Microstructure Technology (IMT), Karlsruhe Institute of Technology(KIT), Hermann-von-Helmholtzplatz 1, Eggenstein-Leopoldshafen 76344, Germany*

**S1. Performance Comparison with typical works of metasurface achromatic imaging**

To more clearly demonstrate the main performance of achromatic metasurfaces in recent years and summarize the development trends in this field, this article reviews the core parameters and design methods of some typical works utilizing metasurfaces for wideband high-resolution imaging, as shown in Table S1.

| **Ref. No.** | **Bandwidth (nm)** | **Aperture (μm)** | **NA** | **Resolution (lp/mm)** | **Achromatic design method** |
| --- | --- | --- | --- | --- | --- |
| [1] | 400~600 | 50 | 0.106 | 228 | Ideal wideband phase matching |
| [2] | 470~670 | 220 | 0.02 | ≈ 40 | Ideal wideband phase matching |
| [3] | 400~660 | 21.65 | 0.216 | 256 | Ideal wideband phase matching |
| [4] | 650~1000 | 30 | 0.24 | 228 | Ideal wideband phase matching |
| [5] | 460~650 | 30 | 0.155 | 228 | Ideal wideband phase matching |
| [6] | 400~800 | 20 | 0.5 | 80.6 | Multi-layer topology optimization |
| [7] | 400~1000 | 50 | 0.164 | 57 | Asymptotic phase compensation |
| [8] | 1000~1800 | 300 | 0.02 | 40.3 | Phase dispersion compensation |
| [9] | 400~700 | 400 | 0.02 | 57 | Dispersion-matched layers |
| [10] | 450~650 | 1e4 | 0.25 | ≈ 113 | End-to-end learning reconstruction |
| [11] | 400~1100 | 1e4 | 0.1 | ≈ 150 | Frequency-domain coherence optimization |
| [12] | 400~800 | 1e5 | 0.24 | 181 | Multi-level structure inverse design |
| [13] | 400~700 | 200 | 0.45 | ≈ 250 | Cubic phase for extended-depth-of-focus |
| [14] | 450~700 | 350 | 0.2 | ≈ 50 | Vortex Bessel beam imaging |
| **Our work** | **450~700** | **4e3** | **0.067** | **244** | **Multi-field Bessel beam imaging** |

Table S1. Main performance comparison for metasurface achromatic imaging research.

Ref. [1–5] focus on the most essential achromatic method for metalenses, i.e., achieving phase matching across a continuous spectrum via phase dispersion engineering. Under this framework, increasing the aperture and NA of a metalens requires a significant enhancement of the group delay range provided by the meta-atom library. Such a theoretical demand is extremely difficult to meet with current micro/nano-fabrication capabilities. Consequently, the apertures and NAs of these achromatic metalenses are generally small. In this context, several design approaches involving multilayer and multilevel structures have been proposed. By relaxing the requirements on focusing efficiency and resolution, the stringent constraints on aperture and NA can be alleviated ^[6–9]^. As research on large-aperture achromatic metalenses progresses, the focusing behavior of these elements commonly shifts from point focusing to line focusing, where energy spreads extensively along the optical axis. In this way, for any wavelength within the operational spectrum, the intensity distribution in the focal plane always exhibits a pronounced maximum at the center ^[10–12]^. However, the PSF in this case becomes globally dispersed, making it necessary to employ backend modules, such as image reconstruction algorithms or deep learning, to achieve high-fidelity image restoration.

This logical progression naturally extends to computational imaging based on extended depth of focus. Ref. [13] represents an early seminal work in this regard, while both Ref. [14] and our work employ the Bessel beam, an extended-depth-of-focus light field distinguished by its superior wideband consistency. After performing highly noise-resistant image restoration, our work achieves an angular resolution test of 5.13 lp/mrad, corresponding to a spatial resolution of 244 lp/mm. This performance reaches approximately 80% of the diffraction limit of a 4-mm-aperture lens, representing a competitive level among works employing millimeter-scale metasurfaces for non-strictly achromatic imaging.

Furthermore, structural designs for achromatic metasurfaces that specifically optimize for off-axis field imaging performance remain scarcely reported. Designs focusing solely on the on-axis field are reasonable for metasurfaces with extremely small apertures. However, as the aperture increases to millimeter or even centimeter scales, the impact of off-axis aberrations becomes comparable to that of chromatic aberrations. Accordingly, a meta-axicon design method capable of correcting both lateral chromatic aberration and off-axis aberrations is conducted in this paper, thereby demonstrating significant advantages in off-axis field imaging as well.

**References for this section**

[1] Wang, S. M. et al. A broadband achromatic metalens in the visible. *Nature Nanotechnology* **13**, 227-232 (2018).

[2] Chen, W. T. et al. A broadband achromatic metalens for focusing and imaging in the visible. *Nature Nanotechnology* **13**, 220-226 (2018).

[3] Lin, R. J. et al. Achromatic metalens array for full-colour light-field imaging. *Nature Nanotechnology*, vol. **14**, 227-231 (2019).

[4] Wang, Y. J. et al. High-efficiency broadband achromatic metalens for near-IR biological imaging window. *Nature Communications* **12**, 5560 (2021).

[5] Zhang, Y. et al. On-chip integration of achromatic metalens arrays. *Nature Communications* **16**, 7485 (2025).

[6] Pan, C. F. et al. 3D-printed multilayer structures for high-numerical aperture achromatic metalenses. *Science Advances* **9**, eadj9262 (2024).

[7] Hu, Y. Q. et al. Asymptotic dispersion engineering for ultra-broadband meta-optics. *Nature Communications* **14**, 6649 (2023).

[8] Balli, F. et al. A hybrid achromatic metalens. *Nature Communications* **11**, 3892 (2020).

[9] Chang, S. Y. et al. Achromatic metalenses for full visible spectrum with extended group delay control via dispersion-matched layers. *Nature Communications* **15**, 9627 (2024).

[10] Fröch, J. E. et al. Beating spectral bandwidth limits for large aperture broadband nano-optics. *Nature Communications* **16**, 3025 (2025).

[11] Xiao, X. J. et al. Large-scale achromatic flat lens by light frequency-domain coherence optimization. *Light: Science & Applications* **11**, 323 (2022).

[12] Majumder, A. et al. Color astrophotography with a 100 mm-diameter f/2 polymer flat lens. *Appl. Phys. Lett.* **126**, 051701 (2025).

[13] Colburn, S. et al. Metasurface optics for full-color computational imaging. *Science Advances* **4**, eaar2114 (2018).

[14] Du, X. M. et al. Full-Color Quasi-Achromatic Imaging with a Dual-Functional Metasurface. *Nano Letters* **25**, 8143-8150 (2025).

**S2. Simulation of Bessel beam intensity**

This study employs COMSOL Multiphysics 6.0 optical simulation software to conduct complex transmittance simulations of meta-atoms and diffraction efficiency simulations of localized grating structures. For the meta-atom complex transmittance simulation, the geometric model is illustrated in Fig.S1(a), featuring perfect matching layers at the top and bottom boundaries, with periodic boundary conditions applied to the sidewalls. A plane wave with 532 nm design-wavelength is incident from the quartz glass substrate port, while the output port is positioned in the near-field region immediately above the Si_3_N_4_ nanopillar to detect the complex amplitude average of the scattered field, thereby obtaining complex transmittance information for meta-atoms with varying cross-sectional dimensions.

Due to the constant phase gradient along the radial direction of the meta-axicon, its overall diffraction efficiency can be approximately considered equivalent to that of a meta-grating with the same phase gradient. Therefore, a simulation model for a single period of the meta-grating was established to calculate its wideband focusing efficiency, as shown in Fig. S1(b). After extracting the Fourier characteristics of near-field information, the diffraction efficiency curve (blue solid line in Fig. S1(c)) can be obtained, which represents the ratio of the 1st-order diffracted intensity to the incident intensity. Due to the higher near-field transmittance of the designed structure at the long-wave end, the transmittance spectrum slightly shifts from the design wavelength towards the long-wave end. On the other hand, for a fixed focal plane *F_B_* = 21mm, the relative position of the focal spot in the finite-length Bessel beam is different, leading to a deviation in the relative intensity distribution of the Bessel spot from the diffraction efficiency distribution. Based on the known first-order diffraction intensity, the relative intensity curve of the Bessel spot (orange dashed line in Fig. S1(c)) can be obtained through far-field simulation of the modulated light field, serving as a reference for intensity calibration of the RGB tri-channels in the imaging process.

The far-field intensity simulation method is provided by the scalar diffraction formula based on angular spectrum propagation theory, as shown in Eq. (S1), where *λ* represents the wavelength, *z* denotes the propagation distance, and ${\tilde{\text{E}}}_{\text{1}}\text{(}\text{x}\text{, }\text{y}\text{)}$, ${\tilde{\text{E}}}_{\text{2}}\text{(}\text{x}\text{, }\text{y}\text{)}$ represent the optical field distributions at the near-field of the metasurface and at the focal plane, respectively. *f_x_* and *f_y_* are the spatial frequencies corresponding to the *x* and *y* spatial coordinates, respectively. ℱ and ℱ ^-1^ represent the Fourier transform and its inverse transform, respectively.

|  | $\tilde{E}_{2}\text{(}\text{x}\text{, }\text{y}\text{)}\text{ =}\mathcal{F}^{-1}\left\{ \mathcal{F} \left[ {\tilde{\text{E}}}_{\text{1}}\text{(}\text{x}\text{, }\text{y}\text{)} \right]\text{}\text{exp}\left( \frac{\text{2π}\text{i}}{\text{λ}}\text{z}\text{}\sqrt{\text{1-}\left( \text{λ}\text{f}_{\text{x}} \right)^{\text{2}}\text{-}\left( \text{λ}\text{f}_{\text{y}} \right)^{\text{2}}} \right) \right\}$ | (S1) |
| --- | --- | --- |


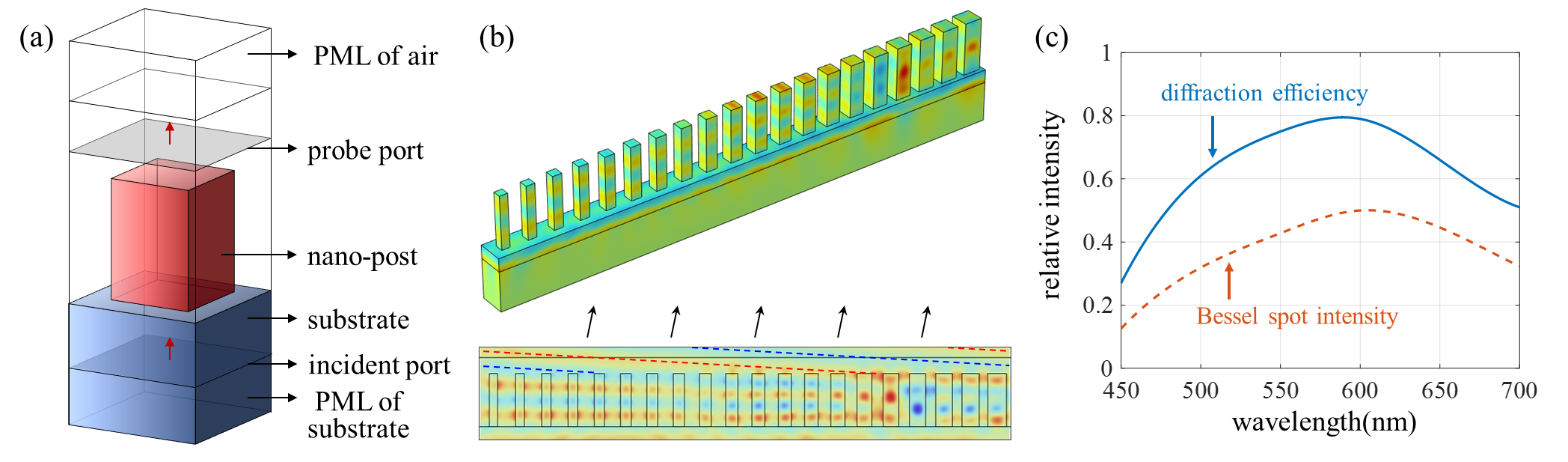


Fig. S1. (a) Schematic diagram of the meta-atom simulation model; (b) 3D surface and cross-sectional plots of the complex amplitude distribution (real(***E_x_***)) of the periodic grating structure; (c) Diffraction efficiency curve and spot relative intensity curve.

It is worth noting that the 1st-order diffraction efficiency is used to describe the energy utilization ratio of the meta-axicon in this work, rather than adopting the evaluation metric commonly employed for most point-focusing metalenses, which only considers the central energy proportion of the PSF (e.g., energy within 3 or 5 times the FWHM). This is because, for the image restoration module, all diverging energy constitutes effective light, not merely the energy near the main lobe. The main lobe energy of the Bessel beam may only account for about 0.1% of the total energy, thus, using this data to evaluate the energy utilization efficiency is meaningless.

**S3. Phase distribution design of wideband off-axis meta-axicon**

To modulate obliquely incident plane waves into off-axis Bessel beams while minimizing chromatic aberration, an off-axis meta-axicon suitable for the visible spectrum has been designed and used to assist the main meta-axicon in FOV expanding. The following is a detailed calculation method and main parameters for the phase of the off-axis meta-axicon:

1. Determine the central wavelength *λ*_0_ of the off-axis meta-axicon to be 532nm, the off-axis focal length *F*_0_ to be 30mm, the back working distance *F_B_* to be 21mm, the aperture angle *α*_0_ to be 2.86°, (corresponding to an approximate aperture of 2*F*_0_tan*α*_0_ =3mm), and the design field angle *θ* to be 4°.
2. To achieve a wideband achromatic design, the optimal tilt angle for the emitted Bessel beam can be solved as *θ'* = *θ* - 0.105mrad according to Eq. (6) and Eq. (7) in the main text.
3. As shown in Fig. 3(a) of the main text, a conical surface is constructed with the off-axis focal point (0, *F*_0_tan*θ'*, *F_0_*) as the vertex and *α*_0_ as the semi-vertex angle. The intersection of this conical surface and the xoy plane is the contour Г of the off-axis meta-axicon, whose analytical equation is shown in Eq. (S2). The parameters *a*, *b*, and *d* are given in Eq. (S3).

|  | $\frac{x^{2}}{a^{2}}+\frac{{(y+d)}^{2}}{b^{2}}=1$ | (S2) |
| --- | --- | --- |
|  | $\begin{matrix} a=\frac{F_{0}\tan\alpha_{0}}{cos\thetaʹ\sqrt{1-{d^{2}}/{b^{2}}}} \\ b=\frac{F_{0}}{2}[\tan\left( \thetaʹ+\alpha_{0} \right)-\tan\left( \thetaʹ-\alpha_{0} \right)] \\ d=\frac{F_{0}}{2}[\tan\left( \thetaʹ+\alpha_{0} \right)+\tan\left( \thetaʹ-\alpha_{0} \right)-2\tan\thetaʹ] \end{matrix}$ | (S3) |

1. For any point (x, y) within the elliptical contour Г, the conical surface where the traced ray lies is linearly scaled with respect to the outermost conical surface, with a scaling factor *K* ∈ [0, 1]. By replacing *a*, *b*, and *d* in Eq. (S2) with *K*⋅*a*, *K*⋅*b*, and *K*⋅*d*, respectively, the corresponding *K* value for that point can be obtained, as shown in Eq. (S4).

|  | $K=\frac{yd+\sqrt{{(yd)}^{2}+(b^{2}-d^{2})(y^{2}+{x^{2}b^{2}}/{a^{2}})}}{b^{2}-d^{2}}$ | (S4) |
| --- | --- | --- |

1. After knowing the scaling factor *K* at any given point (x, y), the central region of the meta-axicon where *K* < 0.5 is considered to have no contribution to the light intensity at the imaging position and is set as an opaque structure. For the region where *K* ≥ 0.5, the phase required at each point to generate an off-axis Bessel beam can be solved based on the principle of equal optical path length, as shown in Eq. (S5). This equation is modified from Eq. (5) in the main text, where *θ* at other positions except for the incident tilt term has been replaced with *θ'* to achieve wideband chromatic aberration suppression.

|  | $\frac{2\pi}{\lambda_{0}}ysin\theta+\Phi+\frac{2\pi}{\lambda_{0}}\sqrt{x^{2}+{(KF_{0}tan\theta'-y)}^{2}+{(KF_{0})}^{2}}=\frac{2\pi}{\lambda_{0}}\frac{KF_{0}}{cos\theta'}cos\alpha_{0}$ | (S5) |
| --- | --- | --- |

The ultimately solved phase of the off-axis meta-axicon visually resembles an on-axis axicon, but there are subtle differences in terms of effective aperture and wavefront aberration, as shown in Fig. S2(a). Additionally, a wideband achromatic off-axis meta-axicon was designed for light incident at 20° to validate the accuracy and applicability of the off-axis meta-axicon design method under large incident angles. In practice, however, the number of meta-axicons increase nonlinearly and rapidly as the requirement of the FOV increase. Developing a meta-axicon cluster with a half field angle of 20° requires careful consideration of how to reduce the number of meta-axicons to achieve a compact design—an issue worthy of exploration in future research.


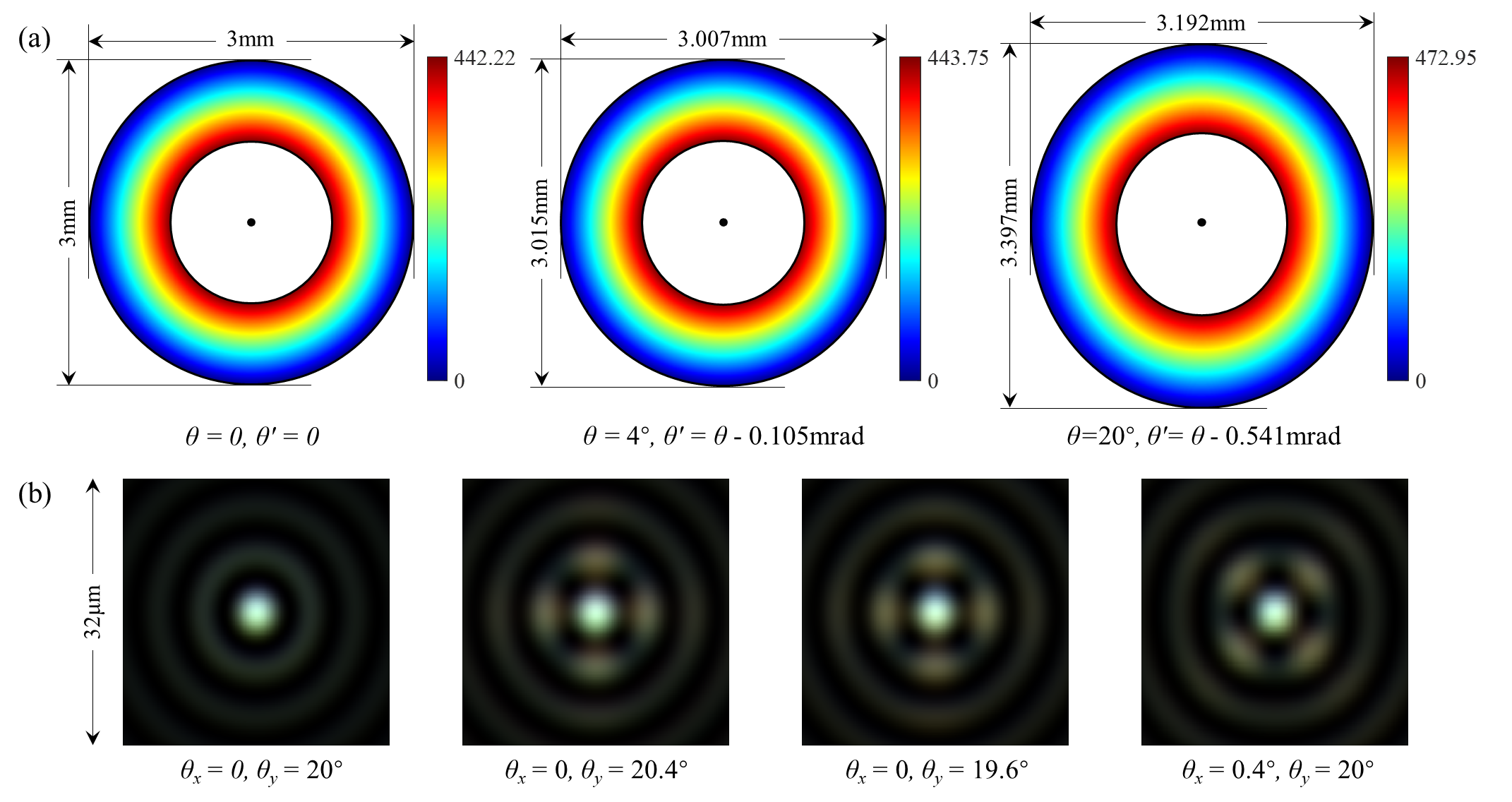


Fig. S2. (a) Schematic diagram of the phase of the off-axis meta-axicon designed with incident angles *θ_y_* of 0°, 4°, and 20°, with the colorbar unit being radians; (b) Bessel spot of the meta-axicon designed with an incident angle *θ_y_* of 20° under a field angle deviation of 0.4°.

**S4. Full-wave simulation and wide-spectrum consistency verification of global PSF**

In this work, the convolution kernel of the imaging system is Bessel light, which exhibits significant intensity divergence characteristics compared to conventional point-focused imaging. Theoretically, the wideband consistency test of the PSFs should not be limited to the central region of the Bessel spot, but rather requires comprehensive testing and analysis of the global dispersion characteristics of the PSFs. To this end, the experiment focuses on the intensity distribution detection of the global PSF for the main meta-axicon. The logarithmic intensity maps under RGB tri-channels are shown in Fig. S3(a), and the corresponding simulation reference image is shown in Fig. S3(b). Both the measured and simulated ranges are 4mm×4mm, which is the same width as the aperture *D*_1_ of the main meta-axicon.


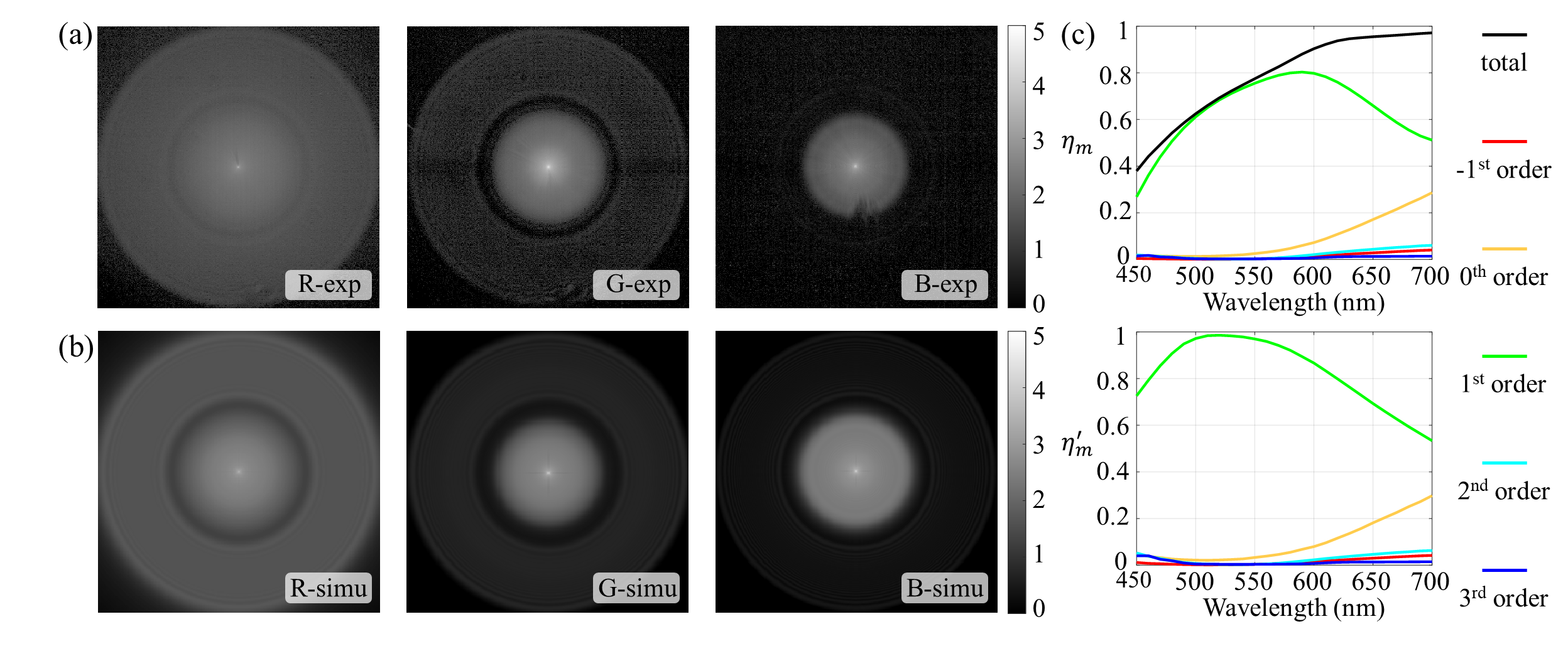


Fig. S3. (a)(b) Measured and simulated results of the logarithmic intensity (log_10_ *I*) of the global PSFs in RGB tri-channels; (c) Graphs of total diffraction efficiency and transmission field diffraction efficiency used to assist multi-order global simulation.

The results in Fig. S3 show that the global PSF is primarily composed of Bessel light from the 1st-order diffraction and direct transmitted light from the 0th-order diffraction. These two components are spatially separated, occupying the regions where *r* < *D*_1_/4 and *D*_1_/4 < *r* < *D*_1_/2, respectively. During the actual measurement process, equal gains were maintained for RGB tri-channels. The 16-bit depth camera used is theoretically capable of reflecting the logarithmic intensity difference of log_10_(2^16^)≈4.8. To prioritize ensuring that the central intensity response remains unsaturated, the 0th-order diffraction in blue channel is indistinguishable from background noise due to its excessively low intensity.

This work also establishes a corresponding simulation process as a reference for the measured results. To achieve equivalent full-wave simulation at the global scale while avoiding massive consumption of computational resources, the local grating approximation described in **S1** was still adopted. Specifically, the Fourier characteristics of the grating structure in Fig. S1(b) are regarded as those of the global structure. Therefore, the transmitted light field of the global simulation can be decomposed into the superposition of conical waves of multiple diffraction orders, as shown in Eq. (S6). Here, *P* represents the grating period, and $\tilde{c}_{m}$ denotes the Fourier coefficient of the *m*-th order. The coefficients are obtained by projecting the simulated outgoing field $\tilde{E}_{P}$ extracted within a single grating period onto the *m*-order plane-wave basis vectors, as shown in Eq. (S7).

|  | ${\tilde{\text{E}}}_{\text{1}}\text{(}\text{r}\text{)=}(r\geq\frac{D_{1}}{4})\left( r\leq\frac{D_{1}}{2} \right)\sum_{m=-\infty}^{\infty} \tilde{c}_{m}exp(i2m\pi\frac{r}{P})$ | (S6) |
| --- | --- | --- |
|  | $\tilde{c}_{m}=\frac{1}{P}\int_{0}^{P} \tilde{E}_{P} exp(-i2m\pi\frac{x}{P})dx$ | (S7) |

In fact, the diffraction orders of the transmitted light intensity that may fall within the region of interest in Fig. S3(b) are only -1th, 0th, 1st, 2nd, and 3rd. The summation in Eq. S6 is only performed for these five diffraction orders. The intensity spectra of these orders are shown in Fig. S3(c), $\eta_{m}={{|\tilde{c}}_{m}|}^{2}$ represents the total diffraction efficiency under the condition where the incident light intensity is normalized, while the corresponding intensity ratio $\eta_{m}^{'}={{|\tilde{c}}_{m}|}^{2}/\sum_{m=-\infty}^{+\infty} {{|\tilde{c}}_{m}|}^{2}$ represents the diffraction efficiency of the transmitted field.

Overall, the measured PSFs and the simulated reference images exhibit a basic consistency in the intensity distribution trend. Especially for the R- and G- channels, the intensity distributions of 0th-order and 1st-order light are highly similar to the corresponding simulation results. The intensity distribution of 1st-order light in B-channel slightly deviates from the simulation results, which is likely due to the lower process robustness at the short-wave end, leading to actual diffraction efficiency lower than the simulation value. In addition, there is a positive correlation between the intensity of the 0th-order direct transmission and wavelength, with the trend of intensity variation differing from that of the 1st-order. The approximation must be made that the ratio of two diffraction orders within any channel is uniform at different wavelengths, thereby the actual image restoration process based on wideband consistent PSF can be executed. Since the total intensity of the 0th-order diffraction can still be considered much lower than that of the 1st-order and only acts on the edge FOV, it does not seriously affect the overall image restoration results.

**S5. ‌Nano-fabrication technology of the metasurface**

In this work, the metasurface developed consists of Si_3_N_4_ nano-pillars and Cr apertures. The Si_3_N_4_ nano-pillar array has a lattice size of 400nm, a cross-sectional side length ranging from 120nm to 280nm, and a structural height of 800nm. To fabricate this structure, an Oxford SYSTEM 100 Plasma-Enhanced Chemical Vapor Deposition (PECVD) system was first used to deposit 800nm-thick Si_3_N_4_ on a quartz substrate, completing the substrate prefabrication.

For the two-dimensional patterning process of the structure, a highly flexible and precise electron beam lithography (EBL) technique was selected. The EBL equipment (Raith EBPG5200 Plus) has a minimum exposure feature size of 8nm and supports samples ranging from 1 inch to 8 inches. The machine achieves a single-shot writing field of 1mm and a stitching accuracy better than 10nm, which is much smaller than the size of nano structures. It meets the processing accuracy requirements for the nano-pillars while maintaining high processing efficiency. In addition, the structural layouts of multiple off-axis meta-axicons are obtained by rotating and replicating a single layout multiple times around the center. The orientation of the pillar array is divided into two types: 0°/90° and 45°/-45°. During electron beam exposure, we precisely controlled the direction of the electron beam spot to align with the array direction to enhance the smoothness of the structural edges.

To meet the selectivity ratio of dry etching and shorten the exposure time, a positive photoresist exposure process combined with a lift-off process was used to form a metal hard mask. The photoresist selected was Zep520A positive photoresist with a minimum resolution of 30nm. The thickness of the spin-coated electron beam photoresist was approximately 300nm. Subsequently, a 50nm thick Cr mask was deposited using a FHR Boxx magnetron sputtering equipment‌, and the hard mask transfer was achieved through the lift-off process of the electron beam photoresist. In the structural etching process, the deep reactive ion etching (DRIE) process was selected. The etching equipment used was an STS-HRM (B102) model, with an etching uniformity of <±5% and a sidewall verticality of ≥87°. Subsequently, the residual Cr mask was removed to complete the preparation of the nanopillar array.

After the fabrication of nanostructures in the specified region, a Cr aperture was fabricated in the remaining area. Following spin-coating of the ultraviolet (UV) photoresist, UV alignment exposure was performed using a Suss MA6 UV exposure machine. Subsequently, a 100-nm-thick Cr film was deposited by the aforementioned magnetron sputtering equipment. Finally, the Cr aperture was formed via a lift-off process of the UV photoresist. The detailed nano-fabrication process flow of the metasurface is illustrated in Fig. S4.


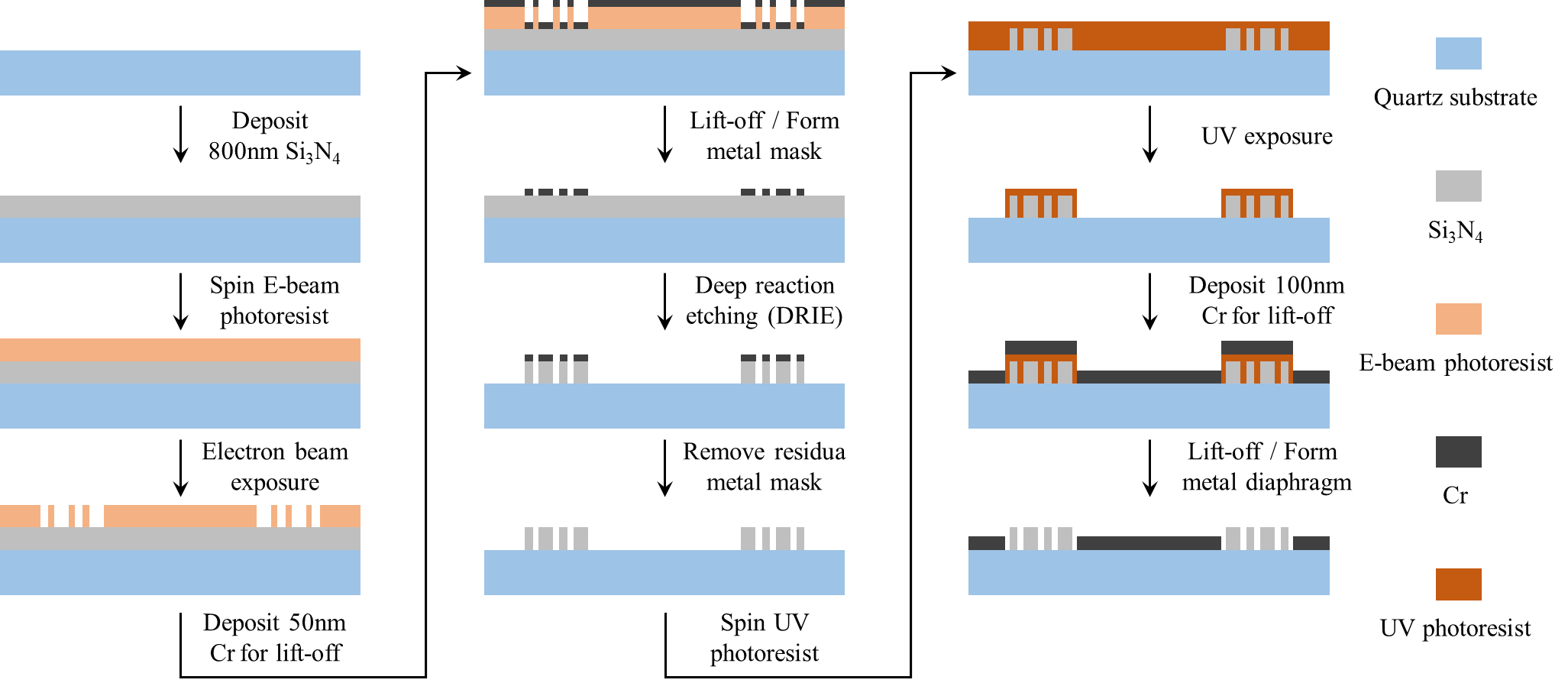


Fig. S4. Flowchart of nano-fabrication process for the metasurfaces.

**S6. Target information for resolution testing**

Various types of resolution targets were utilized in our work to evaluate the resolving capability of the meta-camera. Due to limitations imposed by factors such as target size and the installation adaptability of the beam expansion and collimation system, the standard USAF1951 resolution target was not employed during the testing process. Instead, two distinct customized targets were employed for the test. The design drawings and actual photos of the targets in Fig. 7(a) and Fig. 7(d) of the main text are presented in Fig. S5(a) and Fig. S5(b), respectively. The actual photos of the targets in Fig. 7(g) and Fig.7(h) of the main text is illustrated in Fig. S5(c).

**
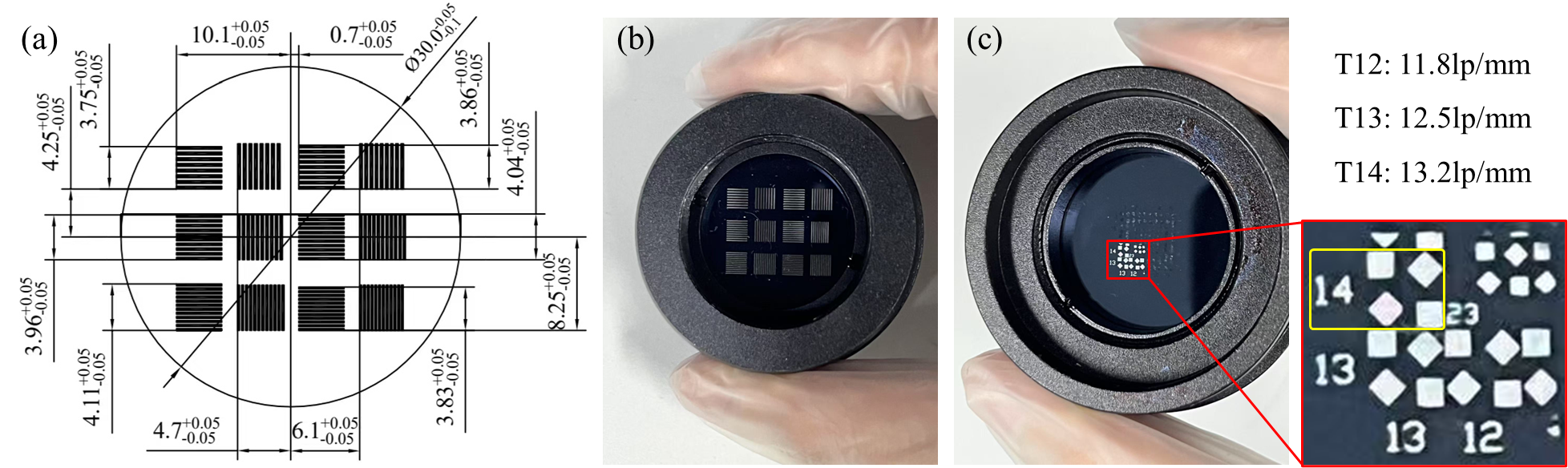
**

Fig. S5. (a) Design drawing and physical image of the resolution target in Fig. 7(a) and Fig. 7(d) of the main text; (b) Physical image of the resolution target in Fig. 7(g) and Fig.7(h) of the main text, and a zoomed-in image of its key observation area.

The targets in Figs. S5(a) and S5(b) were placed at a distance of 1.76m from the metasurface for testing, which contained six pairs of stripes sets T1~T6 with different spatial frequencies. Combining their geometric dimensions and test distance, the angular resolutions of T1~T6 were determined to be: 3.76, 4.10, 4.44, 4.78, 5.13, and 5.52 (unit: lp/mrad). For testing within a small FOV of 0.2°, the pinhole in the beam-expanding collimation system was replaced with a higher-resolution target, serving as an optical equivalent to an infinitely distant object, as shown in Fig. S5(c). The beam-expanding collimation system, as shown in Fig. 5(a) in the main text, has an effective focal length of 0.5m. During the testing process, different regions of the resolution target were sequentially masked, leaving only the specific resolution area under observation to emulate an isolated target within the narrow FOV. Ultimately, ‌the pattern T14‌ was identified as the resolvable limit under these test conditions, corresponding to a spatial frequency of ‌13.2 lp/mm and an angular resolution of ‌6.61 lp/mrad‌.

S7. **Testing and theoretical estimation of depth of field**

In this study, Bessel beams with extended depth-of-focus characteristics contribute to enhancing the imaging depth of field (DOF). While the core design principle of the meta-camera is based on the wideband consistency of diffractive Bessel spots rather than their diffraction invariance over large *z*‑ranges, a large DOF remains a secondary advantage accompanying its achromatic functionality. Here, imaging experiments were conducted with the main meta-axicon on patterns at different object distances. The initial object distance was 80 cm, which was successively halved until reaching 5 cm. The initial width of a single target bar was 3.6 mm and was scaled down proportionally with the object distance. Thus, these patterns share the same angular frequency and produce nearly identical images (note that the patterns were generated by an OLED panel, so the smallest pattern at the 5 cm object distance is affected by pixelation, causing the bar width to slightly deviate from the ideal value). The imaging results are shown in Fig.  S6(a).

**
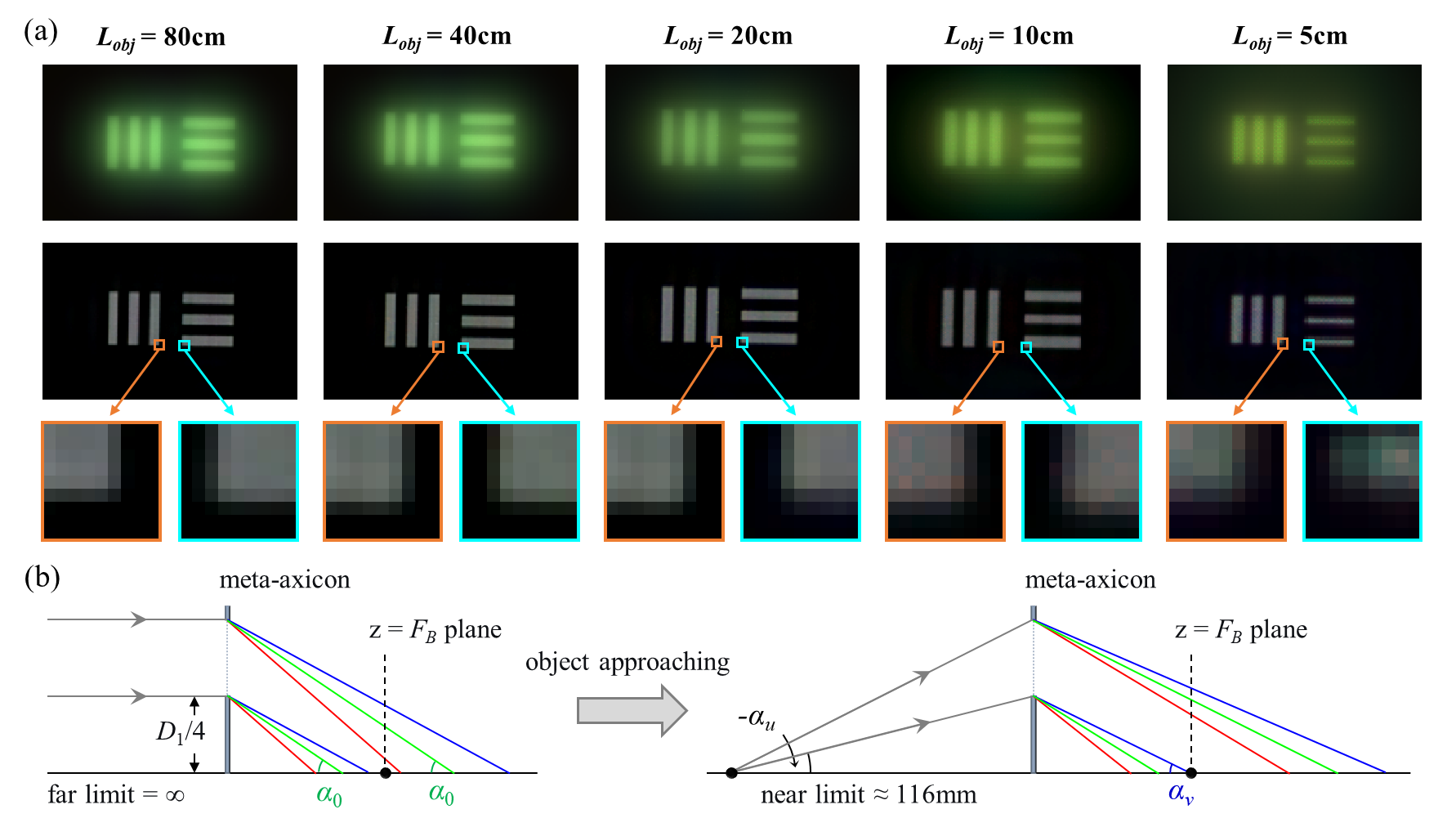
**

Fig. S6. (a) The imaging results of the target patterns under different object distances; (b) Schematic diagram of approximate estimation of depth of field.

The first two rows in Fig. S6(a) show the direct imaging results and the image restoration results, respectively, while the third row visually illustrates the imaging sharpness of the target-bar boundaries. All non‑blind image restoration processes employed a unified convolution kernel, the measured PSF of a point target at infinity, to examine how the output images depend on the object distance. As can be seen from Fig. S6(a), the boundary sharpness shows almost no difference in the first three measurements. A visible decline in boundary sharpness begins to emerge from the fourth measurement and becomes more pronounced in the fifth one.

The above imaging results can be qualitatively interpreted based on the theoretical design: as the object moves from infinity to a closer distance, the beam on the image side correspondingly shifts away from the meta-axicon. In this process, the blue Bessel beam is the first to be completely displaced outside the focal plane, making it impossible to form a blue Bessel spot, as illustrated in Fig. S6(b). At this point, the imaging model significantly deviates from the theoretical basis of image restoration. By establishing a scaling relation determined by the grating equation at the inner aperture of the meta-axicon (*r* = *D*₁ /4), the corresponding DOF limit under this extreme condition can be estimated, as given by Eq. (S8).

|  | $\frac{sin\alpha_{v}-sin\alpha_{u}}{sin\alpha_{0}}=\frac{\lambda_{min}}{\lambda_{0}}, \tan\alpha_{u}=\frac{D_{1}}{4L_{min}}, \tan\alpha_{v}=\frac{D_{1}}{4F_{B}}$ | (S8) |
| --- | --- | --- |

Here, *α_u_* and *α_v_* represent the object-side and image-side aperture angles, respectively, at the near limit of DOF. Based on *λ_min_ =*450nm, *λ*_0_ *=*532nm, *α*_0_ =3.81°, *F_B_* =21mm, the minimum object distance is calculated as *L_min_* ≈116mm. This value closely matches the measured object distance at which the boundaries of the target bars begin to blur. When the object distance falls below this limit, the inadequate image restoration model can no longer produce high‑definition reconstructed images, and chromatic deviations become more pronounced. Although other issues arising from close‑range imaging, such as reduced noise resistance of the blue channel and widened main lobe of the PSF, may also slightly degrade the quality of image restoration, they do not significantly affect the near limit value of DOF. Furthermore, under the paraxial approximation, the same result can be derived for off‑axis meta-axicons with slightly smaller apertures, confirming that the DOF characteristic is uniform across the entire meta-axicon cluster.

Through the dual validation of theoretical modeling and experimental measurements, our meta- camera is ultimately determined to have a near limit of DOF on the order of 100 mm, demonstrating a significantly extended-DOF characteristic compared to conventional near‑diffraction‑limit lenses with the same aperture and NA.
